# Indications and outcomes in bi‐unicondylar knee arthroplasty: A systematic review

**DOI:** 10.1002/jeo2.70266

**Published:** 2025-06-15

**Authors:** Luca Bertolino, Alberto Favaro, Francesco Iacono, Maurilio Marcacci, Tommaso Bonanzinga

**Affiliations:** ^1^ Humanitas University Pieve Emanuele Milan Italy; ^2^ IRCCS Humanitas Research Hospital Rozzano Milan Italy; ^3^ Department of Biomedical Sciences Humanitas University Pieve Emanuele Milan Italy

**Keywords:** arthroplasty, bi‐unicompartmental knee, orthopaedics, osteoarthritis, unicompartmental knee arthroplasty

## Abstract

**Purpose:**

The aim of this systematic review is to analyze and provide an overview of the indications, contraindications and the clinical outcomes to bi‐unicondylar knee arthroplasty (Bi‐UKA).

**Methods:**

A comprehensive search was conducted to identify original studies written in English reporting indication criteria or clinical outcomes on Bi‐UKA) performed simultaneously or at two different stages. Studies reporting patellofemoral implants with medial or lateral implants, ex‐vivo or cadaveric studies were excluded. The study was carried out in accordance with PRISMA guidelines, with the search covering studies up to February 2025.

**Results:**

The literature search identified 783 articles, nine of which were included in this review. A total of 343 patients were identified, of which 257 patients underwent bi‐UKA. Medial and lateral osteoarthritis (OA) are the main indications for Bi‐UKA. The condition of the anterior cruciate ligament (ACL) was taken into consideration by eight studies and 49 patients (19.02%) presenting functional or macroscopic intact ACL were eligible for Bi‐UKA. Age was considered as an eligibility criterion in one study alone. In all studies, Bi‐UKA led to the improvement of clinical scores compared to their pre‐operative values.

**Conclusions:**

Both staged and simultaneous Bi‐UKA are viable options to treat knee osteoarthritis. However, further research is needed to better investigate bi‐UKA.

**Level of Evidence:**

Level III.

**Abbreviations**
ACL: anterior cruciate ligament
AKSS‐F: American Knee Society Score Functional
AKSS‐O: American Knee Society Score Objective
Bi‐UKA: bi‐unicondilar knee arthroplasty
BMI: body mass index
CMS: Coleman Methodology Score
EQ‐5D‐3L: EuroQol five‐Dimension three‐Level questionnaire
F: female
FJS: Forgotten Joint Score
FU: follow up
HSS: hospital for special surgery
HTO: high tibial osteotomy
KSS: Knee Society Score
LCOA: lateral compartment osteoarthritis
M: male
mCMS: modified Coleman Methodology Score
na: not available
NKSS: New Knee Society Score
OA: osteoarthritis
OKS: Oxford Knee Score
PFJ: patello femoral joint
PRISMA‐P: Preferred Reporting Items for Systematic Reviews and Meta‐Analyses Protocols
PROMs: patient‐reported outcomes measures
ROM: range of motion
TKA: total knee arthroplasty
UCLA: University of California Los Angeles
UKA: unicompartmental knee arthroplasty
VAS: Visual Analogue Scale
WOMAC: Western Ontario and McMaster University Osteoarthritis Index

## INTRODUCTION

In the setting of surgical interventions for severe osteoarthrosis (OA), where total knee arthroplasty (TKA) has long represented the gold standard for restoring knee function, bi‐condylar knee arthroplasty (Bi‐UKA) is among the several alternatives available alongside unicondylar arthroscopy, mono and bi‐compartmental knee replacement and high tibial osteotomy [1, 3, 10, 16, 28, 29, 42]. Differently from TKA, Bi‐UKA specifically consists in the replacement of the articular surfaces of both femoral condyles and the tibial plateau of the knee joint alone, without any involvement/replacement of the posterior patella surface [16]. The bi‐condylar replacement may be achieved either in two steps (staged Bi‐UKA), as an addition of a new UKA [16, 40] in response to advancing OA following a primary unicondylar resurfacing, or as a single intervention (simultaneous Bi‐UKA) on both the medial and lateral condyles [44] in patients with severe combined femoral tibial OA. Aside from OA, Bi‐UKA has been also suggested by some authors for use in other clinical conditions such as valgus or varus knee in patients who have undergone medial or lateral meniscectomy, osteonecrosis of both femoral condyles or tibia [38].

Similarly to UKA, its most distinguishing feature is its conservative approach to spare functioning anterior crucial ligaments (ALCs) which yields better native knee kinematics during flexion, better performance in descending stairs, gait as compared to TKA [17, 18] and to preserve healthy bone tissue [26, 35, 36, 41]. Moreover, from the perspective of the patient's experience, the intervention is less invasive (smaller incision and reduced blood loss), is slightly shorter (in some facilities it may be done in the outpatient clinic), and requires shorter recovery time.

Nonetheless, despite having reached the same level of safety as TKA [12, 26, 35, 36, 41], Bi‐UKA remains only a niche procedure (approximately 5% of all knee surgeries in clinical practice) [12, 28] in advanced OA in favour of TKA. In fact, the placement of the multiple components for both condyles requires higher precision, and thus greater surgical expertise, which may weigh against its larger diffusion in surgical practice. It is expected though that the spreading of robotic surgery may somewhat overcome this obstacle [4, 7, 43]. Newer implant models, materials and alignment techniques are also expected to boost the trend of patients receiving this procedure [4, 32].

While the data in literature on UKA are quite extensive [2, 9, 23, 34], those on Bi‐UKA are much fewer, often provided as subsets of data within studies reporting a collection of several surgical approaches. Moreover, the few works which specifically refer to Bi‐UKA use the acronym Bi‐UKA inconsistently across studies, often using it interchangeably with bi‐compartmental knee (which involves the medial or lateral condyle + the patellofemoral joint), making the data difficult to identify and compare. Accordingly, we sought to provide some clarity on the data available and perform an updated search on the literature [1, 29, 42]. Here, we report our systematic review highlighting indications and contraindications to this procedure, as well as providing an overview of the clinical outcomes and the technique.

## MATERIALS AND METHODS

The current systematic review was carried out according to the Preferred Reporting Items for Systematic Reviews (PRISMA) statement [31].

A single search of PubMed, Embase, and Scopus was completed up to February 2025, entering the following query: *“(knee) AND (arthroplasty OR replacement) AND (bi‐unicompartmental OR bi‐compartmental OR bicompartmental OR bi‐condylar OR bicondylar OR bi‐unicondylar OR bi‐UKA OR bi‐UKA OR bilateral UKA)”*. In consideration of the heterogeneity of terms used across literature upon referring to bi‐condylar knee arthroplasty (both condyles of a same knee with no patellofemoral involvement) [1], we based our query on the definitions for bi‐UKA suggested by Garner et al. as well as and the umbrella terms acknowledged as alternatives to define an intervention on the medial and lateral condyles (Supporting Information: Material 1).

Papers were included for systematic review if they fulfilled the following criteria: (i) original studies reporting indication criteria or clinical outcomes on bi‐unicondylar knee arthroplasty, either performed simultaneously or at two different stages (staged bi‐UKA), (ii) being in English language. Whereas they were excluded if they were ex‐vivo or cadaveric studies, cases reporting revision arthroplasty or patellofemoral implants with medial or lateral implants, and formats other than original studies, thus case reports on single‐patient reviews, letters/editorials, book chapters, and methodological papers.

After the removal of duplicates, titles and abstracts were screened by two independent reviewers who excluded papers that did not match eligibility criteria and retrieved the full version of the remaining ones. The full articles then underwent independent review by the two researchers, who discussed the papers and resolved any disagreements by consensus. Reference lists of selected articles were also checked to identify titles that matched our search criteria missed during the original search. Moreover, sample data were compared in papers from same study groups to avoid duplicate data.

The data extracted included first author, year of publication, number of patients, number of knees, sex, mean age, mean Body Mass Index (BMI), indications, contraindications, surgical approach, associated procedures, clinical outcome measurements, mean follow‐up, failures and survival rate.

The methodologic quality of the studies reviewed was assessed separately by two investigators using the modified Coleman Methodology Score (mCMS) (Supporting Information: Material S1). The Coleman Methodology Score (CMS) is based on 10 criteria and ranging from 0 to 100, where <50 points indicates poor quality, 50–69 points fair, 70–84 points good and 85–100 points excellent robustness [11]. The modified criteria allow better reproducibility and relevance for the purpose of the systematic review. Any discrepancy in the independent assessment was discussed and resolved by consensus.

## RESULTS

**Figure 1:**
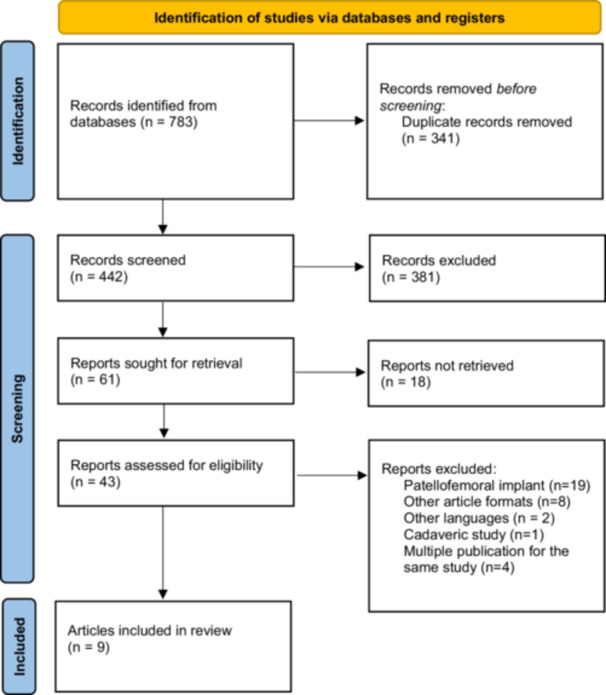
Preferred Reporting Items for Systematic Review and Meta‐Analysis (PRISMA) flowchart for the searching and identification of included studies. PRISMA 2020 flow diagram for new systematic reviews which included searches of databases and registers only.

The literature search yielded 783 articles of which 341 were duplicates. 381 articles were excluded following title and abstract screening. Of the remaining 61 papers, 43 were retrieved and later assessed based on the eligibility criteria, leaving a total of nine articles for systematic review. The screening and review process is summarised in Figure 1.

The mean mCMS score was 52.67 (range 38–75) The average total mCMS and the average mCMS for each criterion are given in Table 1.

**Table 1:** Modified Coleman Methodology Score.

First author	Year	Criteria										
		A1	A2	A3	A4	A5	A6	A7	B1	B2	B3	Coleman score
Banks et al. [5]	2005	0	4	10	10	0	10	0	10	8	5	**57**
Fuchs et al. [15]	2005	4	4	10	0	0	5	5	7	8	5	**48**
Lustig et al. [27]	2008	0	0	10	0	0	10	0	5	8	5	**38**
Parratte et al. [33]	2010	10	7	0	0	5	5	5	7	12	5	**56**
Pandit et al. [32]	2017	0	7	10	0	0	5	0	7	12	5	**46**
Biazzo et al. [6]	2018	4	10	10	0	0	0	0	7	12	5	**48**
Blyth et al. [7]	2021	7	4	10	15	0	10	0	7	12	10	**75**
Garner et al. [18]	2021	7	4	7	10	0	0	0	10	8	0	**46**
Lazzara et al. [21]	2023	4	7	10	0	5	10	0	7	12	5	**60**
**Averages**		4.00	5.22	8.56	3.89	1.11	6.11	1.11	7.44	10.22	5.00	52.67

*Note*: A1: Study size; A2: Mean follow up; A3: Surgical approach; A4: Study type; A5: Description of diagnosis; A6: Description of surgical technique; A7: Description of post‐operatory rehabilitation; B1: Outcome criteria; B2: Procedure for assessing outcomes; B3: Description of subject selection process.

Five [5, 15, 21, 27, 32] reported exclusively data on staged Bi‐UKA (either compared to UKA, compared to pre/post‐operative, or TKA) while two [6, 7] reported on simultaneous Bi‐UKA (Bi‐UKA vs TKA) and two reported both on simultaneous and staged Bi‐UKA (compared to compartmental, or TKA) [18, 33]. Study design and level of evidence are reported in Table 2.

**Table 2:** Study design and level of evidence.

First author	Year	Study design	Level of evidence
Banks et al. [5]	2005	Observational study	II
Fuchs et al. [15]	2005	Retrospective cohort study	III
Lustig et al. [27]	2008	Retrospective cohort study	III
Parratte et al. [33]	2010	Retrospective cohort study	III
Pandit et al. [32]	2017	Observational study without controls	IV
Biazzo et al. [6]	2018	Retrospective comparative study	IV
Blyth et al. [7]	2021	Randomised control trial	I
Garner et al. [18]	2021	Prospective cohort study	II
Lazzara et al. [21]	2023	Case series	IV

### Patient demographics

The characteristics of patient's enroled in the studies reviewed are shown in Table 3. A total of 343 patients with 361 knees were identified. Of these, 257 patients underwent bi‐UKA and 86 TKA. The mean follow‐up was of 68.61 months (range 12–180 months). Among the patients undergoing Bi‐UKA, 50.19% were female. The overall mean age of the complete bi‐UKA cohort being 68.74 ± 5.13 years compared to 67.18 ± 3.77 years for those undergoing TKA.

**Table 3:** Demographic characteristics of patients within the studies reviewed.

First author, year	Surgery	Demographic characteristics											
		Cases		Sex		Age			BMI			Follow up	
		*N*. patients	*N*. knees	M	F	Mean (years)	SD	Range	Mean	SD	Range	*N*. patients	Mean FU, months
Banks et al., 2005 [5]	Bi‐UKA	5	5	3	2	61.8	14.69	NA	NA	NA	NA	5	21.8
Fuchs et al., 2005 [15]	Bi‐UKA	15	15	5	10	67.4	9.4	54–82	NA	NA	NA	15	31.9
Lustig et al., 2008 [27]	Bi‐UKA	6	6	0	6	80	NA	66–85	NA	NA	NA	NA	NA
Parratte et al., 2010 [33]	Bi‐UKA	84	100	33	51	65.7	12.4	32–82	27	3	NA	78	144
Pandit et al., 2017 [32]	Bi‐UKA	25	27	16	9	77.1	6.5	NA	NA	NA	NA	25	48
Biazzo et al., 2018 [6]	Bi‐UKA	22	22	NA	NA	59.7	NA	45–68	NA	NA	NA	19	>180
	TKA	22	22	NA	NA	61.2	NA	48–70	NA	NA	NA	18	>180
Blyth et al., 2021 [7]	Bi‐UKA	34	34	17	17	68.7	7.7	NA	32.4	6.7	NA	32	12
	TKA	42	42	21	21	70.4	7.1	NA	32.6	5.5	NA	39	12
Garner et al., 2021 [18]	Bi‐UKA	22	22	14	8	68	13	NA	28	5	NA	22	17
	TKA	22	22	12	10	67	10	NA	27	4	NA	22	24
Lazzara et al., 2023 [21]	Bi‐UKA	44	44	18	26	74.4	8.4	NA	28.7	5.9	NA	44	84

Abbreviations: Bi‐UKA, bi‐unicondylar knee arthroplasty; BMI, body mass index; F, female; FU, follow up; M, male; SD, standard deviation; TKA, total knee arthroplasty.

### Eligibility criteria/indications for bi‐UKA

The primary indication for which patients in the studies were referred for bi‐UKA were OA of both medial and lateral compartments (simultaneous Bi‐UKA) [6, 7, 15, 33] and progressive OA in the retained compartment after UKA (staged bi‐UKA) [21, 27, 32], and following post‐traumatic bi‐compartmental OA in one study (Table 4) [6, 12]. Knee OA was classified for 144 knees according to the Ahlbäck classification [6, 33].

**Table 4:** Exclusion/inclusion for bi‐UKA.

First author	Indications	Contraindications
Banks et al. [5]	Combined KSS > 195 at 8 months from surgery, return to high levels of activity	NA
Fuchs et al. [15]	Medial and lateral OA; macroscopically intact ACL; circumferences of both legs had to be within 1 cm at the same height; no flexion contracture; knee flexion > 90°; normal adjacent joints and contralateral lower extremity at examination.	Peripheral neuropathy; diabetes mellitus; rheumatological disorders; severe varus or valgus deformity; muscular imbalance in the lower extremities.
Lustig et al. [27]	Elective complaints in compartment opposite to UKA; Stage C or D opposite compartment's knee arthritis; No UKA wear or migration; hypercorrection < 5° of first implant good initial results after UKA; reducibility of frontal plane's deformity; functional central pivot ligaments; near normal ROM; patients; age > 65 years; higher comorbidity; higher risk for TKA.	Absolute: inflammatory arthritis; history of infection; cruciate and/or collateral ligament deficiency; major bone loss; extension deficit > 10°. Relative: associate patellofemoral arthritis; body weight > 80 kg; young and active patients.
Parratte et al. [33]	Medial and lateral OA Ahlbäck Grade ≥ II; preserved PFJ; pre‐operative ROM > 100°; full range of knee extension; clinically stable knee in the frontal and sagittal planes.	Valgus or varus deformity > 108; a planned HTO; a planned or previous ACL reconstruction; a revision arthroplasty; full loss of cartilage on the lateral compartment or a fixed deformity observed on the stress radiograph was considered a contraindication to surgery after 1989
Pandit et al. [32]	End‐stage lateral bone‐on‐bone osteoarthritis progression after medial UKA	Severely damaged PFJ with substantial bone loss laterally. Non‐functional ACL (or a fixed‐bearing lateral UKA depended on the patient's age, health and referred symptoms). Non‐functional ACL with instability.
Biazzo et al. [6]	Medial and lateral OA; asymptomatic PFJ; varus deformity < 8°; BMI < 35; no clinical evidence of ACL laxity or flexion deformity; pre‐operative ROM > 110°.	Medial and lateral OA < Ahlbäck Grade IV; patellofemoral OA < Ahlbäck Grade II.
Blyth et al. [7]	Medial and lateral compartment OA suitable for treatment with a standard unconstrained TKA; clinically intact cruciate and collateral ligaments; patient willing and able to provide informed consent.	Patients with rheumatoid or other inflammatory arthropathy; varus or valgus deformity > 15°; fixed flexion contracture > 10°; single‐compartment OA suitable for an isolated UKA, or radiological evidence of patellofemoral OA > Kellgren and Lawrence Grade III; previous surgery to the knee except arthroscopy; significant OA in the spine or other lower limb joints.
Garner et al. [18]	Not Available	Not Available
Lazzara et al. [21]	Severe symptoms; lateral bone‐on‐bone OA; avascular necrosis of the lateral compartment; a well‐functioning medial UKR.	Not Available

Abbreviations: ACL, anterior cruciate ligament; Bi‐UKA, bi‐unicondylar knee arthroplasty; HTO, high tibial osteotomy; OA, osteoarthritis; PFJ, patello femoral joint; ROM, range of motion; TKA, total knee arthroplasty.

Intact cruciate and lateral ligaments (with no signs of ACL laxity or flexion deformity) was a prerequisite to be considered eligible for Bi‐UKA across all studies [6, 15, 32, 33]. ACL was defined as functional or macroscopic intact in 49 patients (19.07%), making them eligible for bi‐UKA [7, 15]. Cases of non‐functional ACL were reported in one study alone on patients (*n* = 25, 9.73%) with lateral compartment osteoarthritis (LCOA) [32]. The decision on performing a fixed‐bearing lateral UKA (staged bi‐UKA) versus TKA in the presence of a non‐functional ACL was taken intra‐operatively based on the joint's condition after incision, the patient's age, health and referred symptoms; non‐functional ACL correlated to ACL instability definitely contraindicated referral for bi‐UKA [32].

The majority of studies also considered a healthy patellofemoral joint (PFJ) as prerequisite for eligibility to Bi‐UKA [6, 7, 27, 32, 33], excluding patients with PFJ OA scores KL > 3 or Ahlback > II. The study by Blyth with a robotic‐assisted approach allowed eligibility Bi‐UKA with PFJ OA up to KG Grade 3 (Grade 4 were excluded) [7]. However, PFJ symptoms was not considered a specific exclusion criterion for Bi‐UKA [7]. A total of 106 patients (41.25%) were considered eligible for bi‐UKA in the presence of either preserved PFJ [6], asymptomatic PFJ; [6] whereas those presenting severely damaged PFJ [32], PFJ OA [27], PFJ OA classified as Ahlbäck grade greater than II [6] or PFJ OA classified as Kellgren and Lawrence Grade > 3 [7] were not considered suitable for this type of intervention.

Age did not represent a specific eligibility criterion or limitation for undergoing bi‐UKA in favour of another procedure. Only one study from 2008 [27] had specifically excluded patients classified as “young and dynamic” and referred patients to Bi‐UKA if they were 65 years of age or older, reporting an average age of patients who underwent Bi‐UKA of 80 years [27].

### Surgical approaches and implants

The studies reviewed reported both manual placement of components performed by experienced surgeons as well as robotic‐assisted procedures relying on haptic‐controlled passive robotic arm (Table 5).

**Table 5:** Surgical approaches and implants.

First author	Surgical approach	Implant	Associated procedures
Banks et al. [5]	Tibial prostheses were implanted in 2–3 varus with respect to the tibial mechanical axis. Femoral prostheses were positioned perpendicular to the tibial implants with resurfacing bone preparation. The medial and lateral femoral components were lateralized slightly to maintain contact on the centre of the tibial bearing surface with flexion and endo/exo‐rotation	Cemented metal back Fixed‐bearing tibial baseplate and cemented chrome‐cobalt femoral prothesis (Allegretto Zimmer, Switzerland)	Not specified
Fuchs et al. [15]	Medial parapatellar approach	Cemented sledge metal backed prosthesis (seven—Endo, Link, Hamburg, Germany; eight—Search, Aesculap, Tuttlingenm, Germany)	Not available
Lustig et al. [27]	Previous UKA was opened and extended incision after proper lengthening. Then, standard lateral or medial approach	After 1988, fixed all poly‐bearing tibial component.	Not Available
Parratte et al. [33]	Standard medial parapatellar approach for 70 knees and a standard subvastus approach in the remaining 30 knees	Before 1989: Zimmer Condylar II (Warsaw, USA); Alpina (Biomet, Bridgend, UK); after 1989 Miller‐ Galante (Zimmer) cement metal‐backed for UKA	Not available
Pandit et al. [32]	Previous medial UKA was opened and extended, lateral parapatellar arthrotomy	Oxford UKA (Zimmer, UK) Fixed‐baring Miller Galante	Not available
Biazzo et al. [6]	Not available	UC‐Plus Solution (Smith and Nephew, Memphis, USA) with fixed all poly tibial component. Three last patients implanted with fixed metal backed tibial component GMK‐Uni (Medacta, Castel San Pietro, Switzerland).	Avulsion fracture fixation
Blyth et al. [7]	Robotic via MAKO (Stryker). Medial parapatellar incision and approach. The pins used for the navigation arrays were incorporated within the initial incision. Kinematic alignment	Restoris MCK (MultiCompartmental Knee) fixed‐bearing onlay implant	Not available
Garner et al. [18]	Not available	Not Available	Not available
Lazzara et al. [21]	Medial UKA incision was opened and extended. Components are positioned anatomically following a kinematic alignment.	Not available	Exchange of the medial tibial bearing, when required

Abbreviation: UKA, unicondylar knee arthroplasty.

The implants most commonly mentioned were fix‐bearing (such as Miller‐Galante) and mobile‐bearing Oxford. Lazzara et al reported on a novel Fixed Lateral OUKR (FLO) that could be used in place of the Oxford model [21].

### Clinical measures and patient‐related outcomes measures

Most studies measured patient reported outcomes (PROMs) including KSS, OKS, WOMAC scores (Table 6) [27], while fewer outcomes related to kinematics and report proprioceptive abilities and gait patterns, walking distance [5, 15, 18]. In all studies where PROMS were reported, these were comparable and, in most cases, better in patients who underwent bi‐UKA vs TKA. The highest improvement in scores was for Knee Society Score (KSS) for knee and function [6, 7, 33], with a 2.10‐fold improvement in the KSS function score and a two‐fold improvement in the KSS knee score reported by 84 patients (32.68%) [33]. PROMS from the study on robotic‐assisted surgery [7] yielded no statistically significant differences in PROMS scores for Bi‐UKA and TKA at 3 and 12 months from surgery. However, patients in the Bi‐UKA group were able to stand and walk sooner during the first week after surgery and reported VAS pain and stiffness scores consistently lower within the six weeks after surgery [7].

**Table 6:** Pre‐operative and post‐operative functional outcomes for bi‐UKA.

First author	Score	Pre‐operative (SD)	Post‐operative (SD)	Delta (fold change)
Banks et al. [5]	Kneeling flexion	Not available	123.00 (14.00)	Not available
	Lunge flexion	Not available	124.00 (12.00)	Not available
Fuchs et al. [15]	VAS	Not available	8.5 (1.7)	Not available
	HSS	Not available	80.4 (14.6)	Not available
	KSS total	Not available	169.7 (27.1)	Not available
	Patella score	Not available	26.8 (4.5)	Not available
Lustig et al. [27]	Not available	Not available	Not available	Not available
Parratte et al. [33]	Not available	Not available	Not available	Not available
Pandit et al. [32]	OKS	26.00 (8.50)	36.50 (9.20)	10.50 (x1.40)
	AKSS‐O	53.40 (24.90)	81.50 (12.70)	28.10 (x1.53)
	AKSS‐F	66.70 (15.80)	73.80 (18.90)	7.10 (x1.11)
	OKS	26.00 (8.50)	36.50 (9.20)	10.50 (x1.40)
Biazzo et al. [6]	KSS knee	43.65 (3.25)	78.30 (4.00)	34.65 (x1.79)
	KSS function	48.45 (3.25)	80.50 (7.00)	32.05 (x1.66)
	GIUM score	Not available	77.40 (5.25)	Not available
	WOMAC pain	Not available	4.00 (1.50)	Not available
	WOMAC function	Not available	7.22 (1.75)	Not available
	WOMAC stiffness	Not available	1.70 (1.00)	Not available
Blyth et al. [7]	KSS knee	41.00 (3.38)	48.00 (3.13)	7.00 (x1.70)
	KSS function	56.00 (8.94)	102.5 (10.95)	46.50 (x1.83)
	NKSS total	102.0 (10.78)	156.0 (12.00)	54.00 (x1.53)
	OKS	19.00 (2.88)	39.00 (3.13)	20.00 (x2.05)
	VAS pain	6.60 (2.20)	1.60 (2.00)	−5.00 (x0.24)
	VAS stiffness	6.60 (2.20)	2.10 (2.30)	−4.50 (x0.32)
	FJS	Not available	19.00 (4.19)	Not available
	EQ‐5D‐3L VAS	72.20 (15.30)	74.90 (22.30)	2.70 (X1.04)
	EQ‐5D‐3L Index	0.44 (0.30)	0.73 (0.32)	0.29 (X1.66)
	UCLA	3.00 (0.56)	5.50 (0.75)	2.50 (X1.83)
	ROM	101.1 (18.70)	107.1 (12.90)	6.00 (X1.06)
Garner et al. [18]	OKS	Not available	40.9 (7.1)	Not available
	EQ‐5D	Not available	0.91 (0.1)	Not available
Lazzara et al. [21]	Not available	Not available	Not available	Not available

Abbreviations: AKSS‐F, American Knee Society Score Functional; AKSS‐O, American Knee Society Score Objective; Bi‐UKA, bi‐unicondylar knee arthroplasty; EQ‐5D‐3L, EuroQol five‐Dimension three‐Level questionnaire; FJS, Forgotten Joint Score; HSS, Hospital for Special Surgery; KSS, Knee Society Score; NKSS, New Knee Society Score; OKS, Oxford Knee Score; SD, standard deviation; VAS, Visual Analogue Scale; WOMAC, Western Ontario and McMaster University Osteoarthritis Index.

### Function and gait

Function, kinematics, gait, walking and stance, were reported by three studies [12, 28, 29]. Banks which investigated in vivo motions in patients with optimally performing Uni and Bi‐UKA (KSS > 195), reported there were no statistically significant differences in kinematics between Bi‐UKA and UKA for kneeling and lunge, despite kinematic differences consistent with bicompartmental disease and loss of normal lateral feature in Bi‐UKA knees, compared to UKA knees (mean maximum flexion for Bi‐UKA of 123 ± 14 (108–136) and 124 ± 12 (107–135)) [28]. The study by Fuchs [29] on simultaneous implant of sledge protheses, which confirmed there were no significant differences in clinical scores (VAS, HSS, PS and KSS) between UNI and Bi‐UKA, also reported no differences in balance (sway) or gait (knee extension, flexion, stride length), concluding that close to normal joint motion and gait patters observed in Bi‐UKA are likely due to preserved ACL as observed for UKA.

### Follow up and Implant survival

Short‐term follow ups (1–5 years) were reported by most studies reviewed and were between 95 and 100% [21, 33], while two studies alone provide long‐term implant survival data [6, 33] (Table 7). Biazzo et al., reported on a median follow up of 15 years on patients who underwent simultaneous Bi‐UKA from 1998 to 2003 [6, 12]. While the original study had reported statistically significant superior function and stiffness for Bi‐UKA (respectively *p* < 0.05 and (*p* < 0.001) versus TKA immediately after Bi‐UKA surgery, the long‐term follow‐up gave similar results for Bi‐UKA and TKA population, with no statistically significant difference [6]. Paratte et al. provide the survival analysis [33] in a retrospective analysis of cases with a fixed bearing bi‐UKA, observed over a 17‐year follow up period 1972–2000. The reported survival rate was of 78% at 17 years. A total of 17 knees underwent revision surgery after a mean of 6.5 years: 16 for aseptic loosening and one for symptomatic progression of OA in the patellofemoral compartment. Aseptic loosening cases required conventional posterior stabilised TKA with a tibial stem or hinged prosthesis, whereas the knee revised due to OA progression in the PF compartment was treated with the addition of a patellofemoral prosthesis.

**Table 7:** Implant survival.

First author	*N*. Patient at follow‐up	Survival	Failures
Banks et al. [5]	5	Not available	Not available
Fuchs et al. [15]	15	Not available	Not available
Lustig et al. [27]	Not available	Not available	Not available
Parratte et al. [33]	78	78% (at 17 years)	8 Aseptic loosening of both medial and lateral implant, 5 aseptic loosening of the medial implant, 3 aseptic loosening of the lateral implant and 1 for symptomatic patellofemoral osteoarthritis
Pandit et al. [32]	25	100% (at 5 years)	None
Biazzo et al. [6]	19	95% (at 7 years)	1 Resorption of anterior tibial spine
Blyth et al. [7]	32	97%	1 Complication that needed revision
Garner et al. [18]	22	Not available	Not available
Lazzara et al. [21]	44	97% (at 7 years)	1 Medial bearing dislocation

## DISCUSSION

The present study aimed to identify papers that properly referred to Bi‐UKA [16], and highlight the indications and contraindications associated to clinical outcomes and surgical techniques. Data on this procedure are scarce and difficult to find given the inconsistent terminology among studies (by some authors called by the term of bi‐unicompartmental [1, 21], double UKA [38], bi‐uni [6] or lateral UKA following medial UKA [32]) as highlighted by Garner [16]. Indeed, many papers we screened with titles containing the term Bi‐UKA were misleading as they eventually concerned bi‐compartmental UKA (i.e., involving the condyles plus the patella), providing information on somewhat different techniques and implants. This scarcity of data has recently been confirmed by a few systematic reviews [1, 29, 42]. Therefore, we expanded our search to consider any literature updates published since 2023, broadening our scope to both staged and simultaneous Bi‐UKA, and to kinematic studies.

Overall, our systematic search led to nine qualitatively acceptable papers that appropriately referred to Bi‐UKA (either stages or simultaneous). The review highlighted (i) a heterogeneity of surgical approaches, materials and implants which have evolved over the last decades (most retrospective studies are based populations operated with primary UKA of Bi‐UKA over two decades ago) and (ii) the use of updated or modified versions of outcome measurements, such as the KSS score [30], as well as introduction of new parameters like GIUM (especially relevant when interpreting long time follow up) or gait. Taken together, however, the main finding of the present systematic review is that both simultaneous and staged UKA are valid options to address medial and lateral OA, consistently yielding equivalent better outcomes compared to TKA. Moreover, such results are significantly improved when Bi‐UKA is performed with robotic‐assisted surgery [7].

Although it may be argued that a primary medial UKA increases the risk of revision, in particular, of OA progression on the lateral compartment and thus favours the choice toward a TKA, it should be noted that the percentage is extremely low (data from long‐term registries report 2.3%–2.6%) and can be prevented by avoiding overcorrection [32].

### Indications

From a clinical perspective, bi‐UKA was the intervention of choice in patients with progressive OA in the retained compartment after primary UKA, or with osteoarthritis in both medial and lateral condyles, good range of motion in both flexion and extension, preserved patellofemoral joint and without important varus/valgus deformities, and in patients with comorbidities which thus required a less invasive intervention [27].

A major exclusion criterion that prevented patients from undergoing bi‐UKA in most studies was non‐functional ACL with or without instability [6, 12, 27, 32] since anterior cruciate instability can both directly and indirectly lead to tri‐compartmental knee osteoarthrosis, for which TKA is the preferred approach [13, 14] and mainly since it causes abnormal knee kinematics and this increase risks of surgical failures, such as aseptic loosening of the tibial component [19, 24]. However, Pandit et al. [32], extended bi‐UKA to patients with specific conditions in terms of age, general health and reported symptoms also when a non‐functional ACL was present. They reported implant survival of 100% at 5 years follow‐up [32].

In discussing ACL status, it is noteworthy to mention another study which presented data on Bi‐UKA, but which did not meet our inclusion criteria [38]. The authors had tested a surgical technique of bi‐UKA including a concomitant ACL reconstruction. This is currently the only study in the literature that specifically describes this approach for bi‐UKA but unfortunately it does not provide any patient‐related outcome. Yet, several studies have explored ACL reconstruction in the context of UKA, showing the procedure being able to restore knee stability and potentially improve implant longevity [8, 22, 38]. This rationale holds promise for bi‐UKA as well suggesting that a non‐functioning ACL should not automatically exclude a patient from bi‐UKA. Although additional studies are required to confirm this assumption.

In reference to the PFJ status, although PFJ KL > 3 or Ahlbach > II was an exclusion criterion, it was not so for robotic‐assisted surgery. Post‐operative follow‐up results at three and six months showing the PFJ OA degree of severity (mild, Outerbridge 0–3, and severe Outerbridge 3 or 4) was not associated to any advantage/disadvantage of outcome in favour of either Bi‐UKA or TKA suggest the little or no impact of PFJ status in this type of surgical approach.

In reference to age as a parameter to consider or not patient's eligibility, this was not described by most studies as a determining factor. The weighted mean age of patients undergoing bi‐UKA across the reviewed studies was 68.74 years, higher than the weighted mean age for TKA (67.18 years) [20, 22, 25]. Although some authors considered Bi‐UKA most appropriate vs TKA for specific patient populations who were at higher surgical risk due to comorbidities and limited referral for Bi‐UKA to these patients [27], the safety [32] and good short and long‐term outcomes suggest Bi‐UKA as a suitable option also in younger patients [1, 28, 29, 42].

### Surgical approaches and surgical complications

From a surgical perspective, the procedure used for Bi‐UKA does not differ much from TKA, being a midline cut with a parapatellar approach [12]. During second‐stage UKA, the compartment of interest is approached by opening and extending the primary incision. However, the independent positioning of each UKA increases technical difficulty and requires greater accuracy by the surgeon and the mini‐invasive approach entails a poorer field of vision [16, 37]. Potential sources of error span from an overcorrection of alignment to flexion/tension instability, incorrect implant placement, implant size, ACL injury and excessive slope [37].

The first studies to report on manual positioning of Bi‐UKA date back to approximately twenty years ago describe approaching with an approximately 12–13 cm mid‐patellar skin incision with a single anteromedial arthrotomy and lateral patella retraction. In all cases the medial UKR allowed for correct re‐alignment of the limb by replacing the most severely diseased compartment. The amount of bone to be resected from the medial compartment of the tibia to correct the limb alignment was determined pre‐operatively. Their calculation was based on the amount of axial deformity and the thickness of the implanted components. The minimum tibial bone cut was given by the difference between the prosthesis thickness and the axial deviation angle [12]. Other studies in literature (not included in the review) comment on preference for a tibia‐first technique, using a minimally invasive guide simultaneously in both femoro‐tibial components. The authors stress this critical step of BiUKA in obtaining a correct coronal alignment (anatomical, not mechanical) and recommend performing an under correction of the coronal deformity, so that the coronal orientation of the tibial cuts are perpendicular to the epiphyseal axis of the tibia, respecting height and obliquity of the joint line [38].

One study included on simultaneous Bi‐UKA [6, 12] the authors mentioned two cases of intraoperative fractures of the tibial spine during implantation of the protheses likely cause by excessive intra‐operative traction of ACL despite different slopes on tibial insert, which were solved with intraoperative internal fixation. This stressed the need for the intervention be performed by an expert in joint replacement or employing a computer‐assisted Bi‐UKA [12]. The follow up study on this population reported one case of resorption of anterior tibial spine was seen at 7 years after surgery, which the authors associated to poor balancing of the knee and required conversion to a TKA [6].

Most of the intra‐operative issues or post‐op failures described in the studies above [6], however, appear to have been largely overcome by use of robotic‐assisted technology which allows to perform a comprehensive pre‐operative three‐dimensional planning of knee components (including soft tissue balancing, followed by accurate resection of the femur and the tibia and component placement) on a patient‐specific and disease‐specific basis and allow for further intra‐operative adjustment [37].

Blyth et al. with the robotic‐assisted approach aimed to resurface the medial and lateral compartments, reconstructing each patient's constitutional alignment by manually re tensioning the collateral ligament on the most affected side of the joint (medial collateral ligament for varus OA; lateral collateral ligament for valgus OA). This (but not the trochlea or the patella) was resurfaced without requiring specific ligament rebalancing. Neither the trochlea nor patella was resurfaced. Circumferential denervation of the patella was not performed. The surgeons incorporated pins used for the navigation arrays in the bi‐UKA group within the initial incision. While the authors observed that Bi‐UKA maintains anatomy to a greater extent than TKA, they observed equivalent PROMS and surgical complications immediately post‐operative and at one year were equivalent for both procedures. Moreover, they note the robotic‐assisted procedure was much longer than TKA and was not improved by learning curve [7]. However findings from the Truck study [4, 7] highlight the role of robotic‐assisted Bi‐UKA in maintaining the anatomy of the knee in all three planes as well as being less impactful on the HKAA confirming the findings from previous studies for UKA [4, 7, 20], while being equivalent in the achievement of a biphasic gait pattern as reported by a recent publication by the same research groups [20].

### Clinical outcomes and function

Overall, findings from our systematic analysis confirm that Bi‐UKA maintains the benefits of UKA described in literature [5, 15, 27] and its advantages over TKA [12, 16]. In most studies PROMS scores were either similar (no statistically significant difference) or better than for TKA [12, 16, 18]. Greater improvement with bi‐UKA was observed specifically in terms of flexion contracture, better ROM, alignment, and stability, as well as better functions after bi‐UKA. As reported by most studies, patients who had undergone Bi‐UKA gained a closer to normal gait (preserved extensor efficiency) and better stair climbing and sit‐to‐stand activity, and walked faster (both simultaneous and staged Bi‐UKA) as compared to patients who had undergone TKA; in Garner et al. study [18], patients with Bi‐UKA had weight acceptance force and mid‐stance forces (when quadriceps are active) closer to those of healthy subjects (and required less effort) compared to patients who had undergone TKA (*p* < 0.02 and *p* < 0.001). Bi‐UKA patients had greater anterior‐posterior stability. Steps were 9 cm longer for patients with Bi‐UKA versus TKA (*p* = 0.003), 13 cm longer stride lengths (*p* = 0.048), nearer normal cadence, contact double support and gait cycle time (all *p* < 0.01) versus TKA.

In the TRUCK study with robotic‐assisted surgery patients with Bi‐UKA were able to walk, stand, walk longer distance and took fewer analgesics compared to those in TKA group already at the third day after surgery. Likewise, they also reported slightly better VAS for pain and stiffness scores and PROMS at 3 and 6 months after surgery, versus TKA [7]. Bi‐UKA maintained the natural anatomy of the knee (including coronal joint line obliquity) in all three planes and preserved closer to normal joint kinematics compared to TKA [7]. Subgroup analyses on patient characteristics prior to surgery did not evidence any association with outcome [7]. In general, however, the results in this study did not reach statistically significant difference between Bi‐UKA and TKA, which the authors comment may be linked to the specific implant used.

### Implant survival

Regarding the implants' survival, implants used for Bi‐UKA showed good survival in the short and long‐term despite a small number of cases of failures reported [6, 7]. Moreover, it should be noted that most of the revised prostheses had been originally implanted before 1988 and are no longer in use. Since then, prothesis and materials have significantly evolved offering improved efficacy.

### Future perspectives

Among the several studies reviewed, the study by Lustig et al. [27] has perhaps outlined in most detail the characteristics of the ideal patient for Bi‐UKA. In consideration of this and studies that followed and contributed to the literature with more recent data, eligibility criteria will likely be expanded to conditions that initially had represented reason for exclusion. Following is a proposal of indications and contraindications to BiUKA to consider towards patient selection (Table 8).

**Table 8:** Patient characteristics associated to best outcome with Bi‐UKA (staged or simultaneous) based on studies reviewed.

Indications for Bi‐UKA	Contraindications
**Osteoarthritis (OA)** (This is the most common condition for referral to UKA and bi‐UKA, with some differences described across studies)	
Concurrent medial and lateral tibiofemoral degeneration (Ahlbach ≤ IV), medium or end‐stage arthrosis [6, 15, 18, 27] Progressive OA on the retained condyle after previous UKA on other knee [6, 15, 18, 27] Presence of intra‐articular bicompartmental deformity following tibial plateau fracture [6, 12] Also in presence of severe varus of proximal tibia (to be confirmed by further research) [7] hypercorrection < 5° [27] Functioning first implant [27]	End‐stage knee osteoarthritis, with PFJ involvement (see exceptions below). Varus or valgus deformity > 15° Inflammatory disease of joints/rheumatoid arthritis Loosening and wear of first prothesis [27]
**Not OA‐related**	
Post‐traumatic knee injury not OA related [6, 12]	
**OA with specific ligament conditions**	
Absence of gross deformity or ligamentous deficiency [7] Preserved cruciate ligaments [15, 27, 33] Patients with lateral OA after medial UKA, with non‐functional ACL without ACL instability [32] No clinical evidence of ACL laxity and preoperative ROM > 110° [6, 12]	Serious combined laxity [6, 12]. Patients with lateral OA after medial UKA, with ACL instability [32].
**OA with specific patellofemoral joint conditions** In general, best outcomes are obtained in patients with healthy PFJ, however some exceptions have been described across studies	
Asymptomatic PFJ (should perform quantitative T2 evaluation of cartilage for OA risk) [33] A previous well‐functioning Oxford medial UKA (in these patients wear rates are negligible and loosening is rare) [32] A previous well‐functioning Oxford medial UKA also with full‐thickness cartilage loss on the medial not weight bearing aspect of the lateral femoral condyle [32] A previous Oxford medial UKA also in presence of PFJ damage (which does not appreciably compromise outcome) [32] With robotic‐assisted surgery, also PFG < III [7]	Symptomatic PFJ arthritis, PFJ Ahlbach > III [6, 12] Previous UKA with fixed‐bearing such as Miller‐Galante (PFJ is the most common failure mode in second decade) [32]
	With robotic‐assisted surgery, PFJ > III
**OA in specific age groups** (In general, no age contraindications; specific age groups suggested by reviewed studies are detailed below)	
Younger patients with intra‐articular bicompartmental deformity high demand mobility [12, 27] Younger patients at higher risk for potential revision Older age and non‐orthopaedic comorbidities	None
**OA in non‐orthopaedic comorbidities**	
Patients requiring a less invasive intervention or (i.e., pts with comorbidities) at higher risk with TKA [15] Patients requiring quicker recovery [7] Patients overweight that can lose weight	Patients with obesity with varus knee morphology in osteoporosis

Abbreviations: ACL, anterior cruciate ligament; Bi‐UKA, bi‐unicondylar knee arthroplasty; FJS, Forgotten Joint Score; TKA, total knee arthroplasty.

### Strengths and limitations

Given the large time frame covered by these studies, we must note that the interpretation of results and comparison across studies must be carried out in consideration of the fact that some retrospective studies refer to data from 1970. Since then, surgical approaches and materials have evolved (especially from the 1990s). Moreover, long‐used scores such as the KSS have been revised (the score of which after 2012 was extended beyond 100) [30] while new ones such as the GIUM, or gait measures have been introduced [33].

Moreover, the small number of studies reporting the outcomes of bi‐UKA as well of patients enroled in all the studies poses additional limits to strength of evidence. As evidenced by the modified Coleman methodology score, the quality of the study designs was low, lacking description of post‐operative rehabilitation and the survival on short and long term.

Despite such limitations we believe our review provides an updated view on the topic and critical appraisal of the data on the clinical outcomes that could perhaps encourage the outlining of future guidelines or recommendations for Bi‐UKA as has recently occurred for UKA [39].

## CONCLUSION

The studies in literature reporting on bi‐unicondylar knee arthroplasty (staged or simultaneous medial and lateral mono‐compartmental knee arthroplasty confirm better outcomes compared to TKA in terms of tissue and ligament sparing, shorter postoperative recovery, better knee function, gait, and faster walking as compared to TKA. Data from literature show the improvement of clinical outcomes over the years in parallel with newer materials and implants, as well as refined surgical techniques, advanced approaches such as robotic‐assisted surgery.

## AUTHOR CONTRIBUTIONS

All authors contributed to the study conception and design. Material preparation, data collection and analysis were performed by Luca Bertolino and Tommaso Bonanzinga. The first draft of the manuscript was written by Luca Bertolino and all authors commented on previous versions of the manuscript. All authors read and approved the final manuscript.

## CONFLICT OF INTEREST STATEMENT

The authors declare no conflicts of interest.

## ETHICS STATEMENT

Not applicable for this study.

## Supporting information

Supplementary Material 1.docx.

Supplementary Material 2.docx.

## Data Availability

The data that support the findings of this study are available from the corresponding author upon reasonable request.
