# Supplementary figures and images for: Indications and outcomes in bi‐unicondylar knee arthroplasty: A systematic review
Source: J Exp Orthop. 2025 Jun 15;12(2):e70266. doi: 10.1002/jeo2.70266 (PMC12167629; doi:10.1002/jeo2.70266)

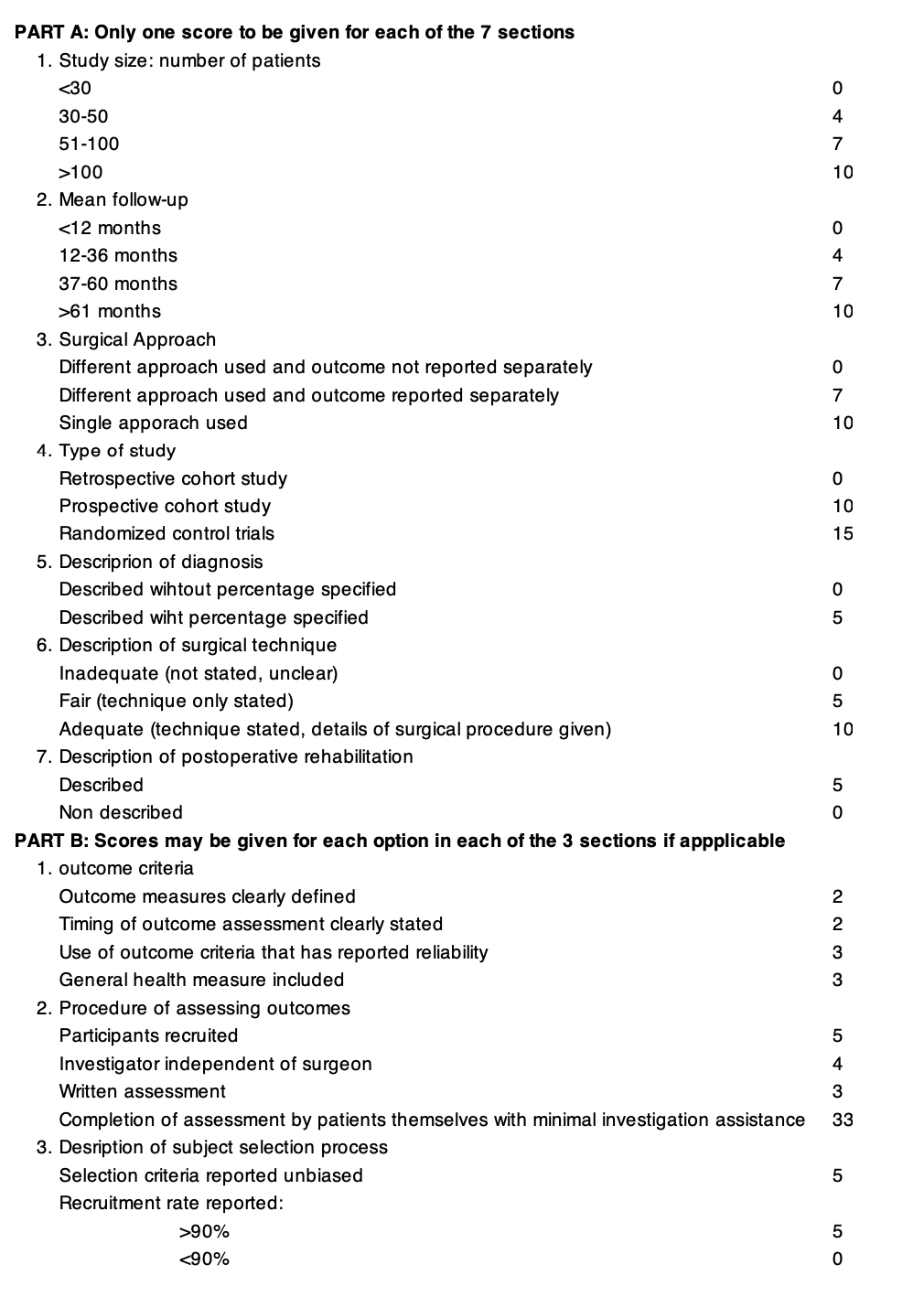

Supplement: Supplementary file 1 — Supplementary Material 1.docx. [file JEO2-12-e70266-s001.docx]
