# Supplementary material for: Indications and outcomes in bi‐unicondylar knee arthroplasty: A systematic review
Source: J Exp Orthop. 2025 Jun 15;12(2):e70266. doi: 10.1002/jeo2.70266 (PMC12167629; doi:10.1002/jeo2.70266)
Supplement: Supplementary file 2 — Supplementary Material 2.docx. [file JEO2-12-e70266-s002.docx]

| DEFINITION | MEDIAL CONDYLE | LATERAL CONDYLE | PATELLOFEMORAL COMPARTMENT | SPECIFIC |
| --- | --- | --- | --- | --- |
| Unicompartimental Knee Arthroplasty (UKA) | X | X |  | Either one of the two |
| Bi-unicompartimental medial | X |  | X | both |
| Bi-unicompartimental lateral |  | X | X | both |
| Bi-unicondyar knee artrhoplasy (Bi-UKA) | X | X |  |  |
| Total Knee Arthroplasty (TKA) | X | X | X |  |

# Bilateral mean UKA (either medial or lateral) in rx and lf knee

# Laterality stands for left and right knee, not condyle
